# Aptamers’ Potential to Fill Therapeutic and Diagnostic Gaps

**DOI:** 10.3390/ph17010105

**Published:** 2024-01-12

**Authors:** Alfredo Berzal-Herranz, Cristina Romero-López

**Affiliations:** Instituto de Parasitología y Biomedicina López-Neyra, Consejo Superior de Investigaciones Científicas. PTS Granada, Av. del Conocimiento 17, 18016 Granada, Spain

More than 30 years ago, in 1990, three independent research groups published several papers demonstrating that genetics could be performed in vitro in the absence of living organisms or cells [1,2,3,4]. This represented a major breakthrough in experimental Molecular Biology, defining the basis for what we now call vitro molecular selection strategies [5]. These strategies allow the selection of nucleic acid molecules, genotype, that can express a specific phenotype on their own. One phenotype that has attracted significant interest and has been widely exploited is nucleic acids’ ability to bind to a specific target molecule, to the extent that the selection of nucleic acids capable of binding specifically and with high affinity to a defined target now constitutes its own dedicated discipline, known as aptamer technology. These efficient nucleic acid binders are referred to as aptamers and the procedure used to select them is known as SELEX, terms coined by Ellington and Szostak [1] and Tuerk and Gold [2], respectively. Aptamers are short RNA or DNA oligonucleotides, and their functionality resides in their three-dimensional structure. Although different procedures have been developed to improve the aptamers’ efficiency and to extend the technical procedure of SELEX [6,7,8,9,10,11,12,13], the technology adheres to the basic principles defined by Gold and Szostak’s research groups back in 1990.

As a result of the thousands of manuscripts published in the past thirty years that describe the selection of RNA or DNA aptamers and their applications, it is widely accepted that they have a broad range of potential applications in different fields of knowledge regarding aptamers technology [14,15,16,17]. Firstly, aptamers are considered excellent candidates for the development of therapeutic molecules to fight different diseases [18,19,20,21,22,23]. Many assays have been performed to investigate their potential to combat infectious diseases caused by RNA viruses, either by targeting a variety of cellular or viral proteins, e.g., [24,25,26,27,28] or by targeting structural elements of the viral RNA genome [29,30,31,32,33,34,35,36,37,38,39]. These publications reported the achievement of a range of therapeutic activity levels in cell culture assays that highlighted the potential of this molecular antiviral strategy. Cancer, in its broadest sense, attracts a great deal of interest and many resources, and aptamer technology is no stranger to this. Cancer treatment has also been the focus of many aptamer-based studies [40,41,42,43,44,45,46]. However, other diseases such as neurodegenerative or cardiovascular diseases, among others, have also been targeted in aptamer studies [47,48,49,50,51,52]. In addition to the development of aptamers as therapeutic molecules, other notable approaches include the applications of aptamers as biosensors, through the development of aptamer-based devices and platforms for disease diagnosis [53,54,55,56,57,58,59,60,61,62,63,64], and as molecular tools to deliver therapeutic drugs to specific target cells, as well as to improve access to malignant molecules whose inactivation is desirable [65,66,67,68,69,70]. These applications have driven the significant development of the aptamers-based discipline in recent years.

In this Special Issue, we have compiled a collection of original articles and reviews focusing on the various factors that define aptamer technology and the main trends in the field. Wrenger and coworkers comprehensively summarize the recent achievements in applying aptamers to the diagnosis of infectious viral diseases, with particular focus on review work applied to RNA viruses such as flaviviruses, influenza virus and coronaviruses [71]. Their review highlights the importance of this discipline aimed at applying aptamers to pathogen detection, which is probably one of the fastest growing applications of aptamers.

Five original research articles included in this Special Issue cover a variety of issues related to using aptamers to treat and diagnose cancer. Dr. González et al. provide evidence of the potential application of selected DNA aptamers, directed against the vaccinia-related kinase 1 (VRK1), as anti-cancer drugs [72]. VRK1 is involved in cell cycle progression [73] and has been linked to the development of several types of cancer [74,75,76,77,78,79,80]. They show inhibition of the cell cycle progression and induction of apoptosis of the MCF7 breast cancer cell line treated with aptamers. Dr. Dubin’s group selects and characterizes DNA aptamers targeting the human programmed death-ligand 1 (PD-L1), whose expression level reflects the immune status of tumors [81]. The assay—both in cell culture of several human tumor cell lines and in two mouse tumor models—of the selected fluorescently labeled aptamers demonstrates their potential for the development of a non-invasive diagnostic method of tumor classification based on PD-L1 status and, therefore, their usefulness for decision-making in personalized immunotherapy. In another interesting article, Dr. Calzada’s group reports on the optimization of the delivery system of an aptamer probe that has already proven to be efficient in the imaging detection of a cancer biomarker. They have developed a device that significantly improves tumor uptake of the aptamer probes in both cell culture and BALB/c mice [82]. The last two articles within this series describe the development of aptamer-based drug delivery devices [83,84]. In their article, Qi and coworkers describe the design of a gemcitabine dendrimer linked to an aptamer specific for breast cancer cells. The aptamer provides the desired cell specificity to deliver the antitumor drug, reducing off-target cytotoxic effects and enhancing specific activity against target cells, improving the therapeutic efficacy of the drug [83]. Heus’ group also designed a smart approach that can be used to improve cell specificity and drug delivery efficiency [84]. They applied an innovative 3D-SELEX strategy against cellular spheroids of breast cancer tumor cells, using non-malignant breast cells as target for counter selection. They demonstrated the specificity of selected aptamers for the breast tumor cells. Further, they designed aptamer multimers which intercalate DNA boxes that efficiently bind and carry doxorubicin, a very potent chemotherapeutic drug. This approach can significantly reduce the undesirable side effects of the drug [84]. 

This Special Issue also includes two original research articles focusing on the applications of aptamers as anticoagulant tools, with demonstrable applications in the fight against ischemic stroke. In the first of these articles, Shea and coworkers characterize the biological activity of a previously described RNA aptamer, BB-031, targeted against the Von Willebrand Factor (VWF), which plays a critical role in thrombosis [85]. Their study uses a microfluidic model of arterial occlusion. They show that BB-031 induces thrombolysis in this model, providing evidence of dose-dependent inhibition of VWF by the aptamer that leads to a reduction in thrombus surface area and recanalization. On the other hand, Dr. Pasternak’s group has addressed the optimization of antithrombin DNA aptamers through the introduction of chemical modifications. In particular, they analyze the effect of pyrrolo-2′-deoxycytidine (Py-dC) and its derivatives modifications. They demonstrate that while all variants tested exhibited anticoagulant activity, a variant with a decyl derivative of Py-dC containing a long, linear aliphatic side chain exhibited greater thrombin inhibition activity [86]. As indicated above, the activity of the aptamers relies on their 3D structure. The antithrombin aptamers optimized by Pasternak and co-workers adopt a G-quadruplex structure, whose thermodynamic stability is preserved with little variation in the chemically modified variants tested. Interestingly, this G-quadruplex structure is shared by other aptamers isolated in independent experiments targeting very different molecules. This is the case for AT11-L0, a DNA aptamer targeted against the nucleolin (NCL), a nuclear protein that plays an important role in angiogenesis in retinal neovascular diseases, since its inhibition promotes an antiangiogenic effect [87]. This aptamer has been used by Moreira and co-workers to functionalize liposomes that could be loaded with antiangiogenetic drugs [88]. The authors show that the complexes allow efficient targeting of NCL, making liposomes functionalized with this aptamer a promising tool to enable effective anti-angiogenic drug delivery. Recent advances in G-quadruplex-mediated cancer therapy are described in [89].

Finally, Dr. Maher III’s group aimed to understand the biological mechanism of a previously described DNA aptamer conjugate with streptavidine, also capable of adopting G-quadruplex structure, shown to promote remyelination in a mouse model of chronic spinal cord demyelination [90]. In their most recent paper, the authors provide evidence that this macromolecular aptamer-based tool binds to the cell membrane of human and adult rat oligodendrocytes, suggesting that binding to a membrane molecule triggers the remyelination pathway [91].

This set of original research contributions providing evidence of the versatility of applying aptamers for the diagnosis or treatment of complex diseases is completed by two very interesting review articles by the groups of Drs. Ilgu and Marty, respectively [92,93]. Dr. Ilgu’s group nicely summarizes the main achievements in the application of aptamers to the treatment or diagnosis of neurological diseases, highlighting the potential future development of the field [92]. Meanwhile, Dr. Marty et al. present a comprehensive review of the current state of the art in biomarker detection using electrochemical aptasensors [93]. The authors provide a very careful summary of disease diagnostic strategies by detecting a variety of biomarkers using an electrochemical aptasensor. They provide examples of biomarkers for the early detection of cancer, heart diseases, degenerative diseases (such as Alzheimer’s or multiple sclerosis), diabetes or infectious diseases. The review also includes very interesting information on the functioning mechanisms and properties of the electrochemical aptasensors. The advantages and future perspectives of aptasensors are also discussed [93]. 

Aptamers, as specific and efficient binders, have inevitably been compared to antibodies in terms of their potential analytical and diagnostic applications. In relation to this, Dr. Bruno has provided a very interesting article in which he discusses situations in which aptamers can fill the gap left by antibodies, indicating that there is no need to compete with antibodies and it is necessary to decide which molecule may be most appropriate for each specific problem [94]. He offers a very interesting reflection on the futility of competing with antibodies and the need to look and identify (using his same words) “niches were aptamers are truly needed or wanted”. Readers can learn a lot from this interesting review article [94]. Continuing this line of investigation, Marty and co-workers provide another review article summarizing the applications of aptamers in the lateral flow assay procedure for rapid detection of different analytes, biomarkers or specific molecules in point-of-care diagnostics. The aptamers are used to substitute the antibodies in this widely used in vitro detection platform, in which several limitations derived from the use of antibodies have been identified. At the same time, the use of aptamers also has some drawbacks that the authors highlight in this article.

The influence of two physical properties of protein ligands, molecular weight and isoelectric point, on aptamers’ affinity has been studied by Fischer and co-workers. Their study including nearly 300 target proteins and peptides spanning a wide range of isoelectric points and molecular weight concludes that there is a significant inverse correlation between the isoelectric point of protein ligands and aptamers’ affinity. In contrast, there appears to be no correlation with molecular mass [95].

In a comprehensive review article, Tickner and Farzan discuss the utility of riboswitches for optimizing adeno-associated viruses (AAV) as transgene vehicles in gene therapy [96]. Riboswitches are structural RNA elements that change their structure as a result of their specific binding of a small molecule, leading to a change in the function of the RNA molecules in which they are contained. Riboswitches constitute an aptamer included in an RNA regulatory element, whose structure is sensitive to aptamer–ligand binding. Original natural riboswitches were identified as regulators of gene expression in bacteria [97,98,99]. Riboswitches have been artificially engineered to develop ligand-responsive regulator devices [100,101,102,103,104,105,106]. The review by Tickner and co-workers provides a detailed description of different types of artificial riboswitches described to date that function in human cells, explaining their mechanisms and their applications in the context of AAV-mediated transgenes delivery [96]. 

The Special Issue concludes with a very interesting article by Andrianova and Kuznetsov, who review a topic of great interest: Biocomputing based on the use of DNA aptamers and their most important feature—their excellent binding abilities—for the creation of logic gates applied to medicine and analytical chemistry [107]. A logic gate performs a Boolean function, a basic logical function that converts the input signals into a logic output. Briefly, an aptamer-based biosensor device constitutes a logic gate that, in the presence of the ligand, generates an output signal. In this review, the authors summarize different biocomputing approaches involving aptamers, describing the various methodologies applied to detect an output signal. They distinguish between optical and electrochemical methods for obtaining an output signal, which are also explained in detail. This represents an innovative and growing application of aptamers, with medicine and analytical chemistry currently being the fields in which it could have the greatest impact. Its development is supported by the continuous identification of new aptamers against their specific ligands.

This Special Issue constitutes a representative sample of reviews and original research articles, providing a snapshot of the current state of the art of aptamers technology. By reading these papers, readers can get a broad idea of the possibilities of use of aptamers, of different strategies for developing tools based on these molecules, and ways to potentially improve their efficacy. This reinforces their potential use in the clinic, which may currently be the most attractive and desired application for aptamer technology.
